# The Institutional Worker

**Published:** 1918-04-20

**Authors:** 


THE
### INSTITUTIONAL WORKER
**Being a Special Supplement to "The Hospital"**

### OUR BUREAU OF INFORMATION.

### Rules for Correspondents.

1. Every letter must be accompanied by the coupon to be cut from the back cover (inside page) of The Hospital, current issue, and must contain the name and address of the correspondent with pseudonym for publication if desired. Replies by post cannot be given save under exceptional circumstances at the Editor's discretion.

2. Letters from Approved Homes in reply to special needs published in the Bureau should state terms and full particulars, and be sent prepaid under cover to the Editor of the Bureau with name written across coupon for identification.

3. Proprietors of Homes which have not yet been entered on the List of Approved Homes, but have spare accommodation likely to suit special needs, are invited to write for an application form for registration. The fee for registration, which includes two announcements of the Home in the Bureau and other privileges, is 10s.

4. All communications to be addressed to the Editor of The Hospital, 28 Southampton Street, Strand, London, W.C. 2, and marked "Bureau of Information."

### INSTITUTION FACTS AND FIGURES.

### How to Sterilise Rubber Gloves.

#### Answer to February Question.

By "Spero."

The question was:

**Describe the best method of sterilising rubber gloves, to be used during operations, to ensure their being perfectly sterilised with the minimum amount of damage to the rubber.**

Having made sure that the gloves are free from holes, put them in a small holland bag, which should have weights sewn in each corner so that it will not float but will become immersed in the water. Drop the bag into a steriliser of *boiling* water, boil for ten minutes (some authorities say five minutes is sufficient), then transfer with sterile forceps to a dish of sterile water. If the gloves are boiled in the same steriliser as that used for instruments, care must be taken that no soda has been put in it or the gloves will become rotten. The water must be boiling when the gloves are put in, so as to allow the maximum amount of heat for the minimum length of time.

If the surgeon wishes his gloves to be dry-sterilised, an ordinary dressing drum or tin about 2 inches longer and wider than the gloves should be obtained. This must be well lined with Gamgee tissue, or lint and wool. Each glove must be well powdered, inside and out, and a piece of lint placed inside each glove, and between each, to keep the rubber surfaces apart. A small dredger of powder may also be wrapped in lint and placed in the drum to sterilise at the same time. The drum is now closed, and the outlets opened, and is then placed in a bag made of lint and wool, or two or three thicknesses of blanket, and the whole is enveloped in a covering of jaconet and put in the steriliser; with steam, a temperature of 248° F. or 15 lb. pressure is necessary for twenty minutes. When sterilised, remove the outer coverings and close the outlets of the drum.

The heavy rubber gloves used for platings are generally placed in Lysol 1 per cent. for two hours or longer, instead of being boiled.

### Repairs and Renewals.

#### Answer to March Question.

By J. R. Y.

The question was:

**Describe the system adopted at your institution for dealing with repairs, including (a) the arrangement in force for the periodical examination of the structure, and (b) the method of collecting, recording, and disposing of repair requisitions.**

The institution with which the writer is connected is a mental hospital with about one thousand patients, and all repairs to the building, engineering plant, furniture, etc., are the special province of the engineer-clerk of works, who is responsible to the medical superintendent for the whole of the artisan staff, a certain sum being provided each year to cover the cost of the workmen's wages and the materials required for the work.

Three requisition papers may be dropped into the clerk of works' letter-box each morning directly after breakfast, according to the repairs required—one from the head attendant for the repairs in the male wards, the second from the head nurse for those in the female wards, and a third from the housekeeper, who is responsible for the central administrative block, kitchen, laundry, and serving-room, these officers having obtained the particulars from those in charge of each ward or department on their early morning round.

A duplicate of these requisitions is kept by the officers for their own reference and that of the medical superintendent.

The clerk of works distributes the items of work, according to their nature, among the various artisans, writing them down on a special board provided for each man, and retaining the requisition papers on three separate files for reference. He also visits the places from whence the more important items of work are reported and supervises their execution.

Examination of the Structure.

This is the constant and especial duty of the clerk of works, as many defects, particularly those noticeable on the outside of the buildings—viz. imperfect slating, decayed woodwork, brickwork, and masonry, leaky gutters and rain-water pipes, overflowing water-cisterns, choked drains, perished paintwork, etc., may escape the attention of the other officers until much harm is done or signs of the damage are shown inside the building.

As, however, there are some portions of the buildings, outhouses, etc., which do not otherwise come regularly to the notice of the clerk of works, he makes a periodical inspection, weekly to some portions, monthly to others, so that no portion of the structure or its contents can be neglected for any length of time.

An item which may also be mentioned is the monthly examination of the fire-escape doors and fire appliances to see that these are in perfect working order.

### APRIL QUESTION.

**A Register of Nurses' Ward Work.**

How should a book be ruled to give at a glance a record of nurses' different duties in male, female, and children's wards, and time spent at classes, etc.? The hospital is a training-school, and nurses take day and night duty. There are three floors for male patients, two for female patients, and a children's block.

### RULES.

The following rules must be observed:—

1. Contributions must be written on one side of the paper. Brevity and terseness are desirable features. The MSS. must bear the name and address of the sender and be accompanied by coupon to be cut from the back cover (inside page) of the current issue of The Hospital. A pseudonym must be chosen if the name is not to be published.

2. Contributions must be addressed to the Editor of The Hospital, Institutional Worker Supplement, 28 & 29 Southampton Street, Strand, London, W.C. 2, should reach him before the end of the current month, and be marked in the left-hand corner "Facts and Figures."

**A minimum payment of Five Shillings will be made for each published answer.**

### QUESTIONS INVITED.

In connection with our Question Box, we offer every month five shillings each for the two best questions which are sent in for consideration. The questions may be on any institutional subject and concern any department, from the secretary's to the porter's, or the matron's to the domestic staff; the one essential is that they must be practical and deal with points that have a definite relation to hospital or institutional administration, upkeep, finance, or management. Points relating to artificers' work, housekeeping, the laundry, out-patients, and other practical matters will be welcome. It is hoped that thus, when difficult or doubtful points arise in the course of their work, institutional workers will be encouraged to put them in the form of a question and send them to the Editor, The Hospital Bureau of Information, 28 & 29 Southampton Street, Strand, W.C. 2, marked "Question Box."

The best questions will be published in due course and our readers will be given the opportunity of answering them. Thus all institutional workers may be able to co-operate with the view of helping the work and smoothing the difficulties of each other. In short, we want the Question Box of the *Institutional Worker* to be freely used by every worker habitually.

### ENQUIRIES AND ANSWERS.

### *THE SICK AND IN NEED.*

**Convalescent Homes.**

We do not know of any suitable institution in the town you mention, but perhaps one of the following may meet your requirements: The Merchant Taylors' Company's Convalescent Home for Ladies (there is also one for men), Bognor; admission is free. You must make your application to the Clerk, Merchant Taylors' Company's Convalescent Homes, The Hall, Threadneedle Street, London, E.C. The Victoria Wellesley Home for Convalescent Women and Girls, Walton Road, Bognor; the terms of admission to this Home are by letter of recommendation, and a small weekly payment. Apply to the Lady Superintendent.—Anchor.

**Home for Incurable Case in Yorkshire.**

St. Catherine's Home for Cancer and Incurables, Bradford, provides for women in the last stages of cancer and other incurable diseases, and we think that this might be a most suitable institution for the case you refer to. Admission is free upon application to the committee.—Elsie.

### *EMPLOYMENT AND TRAINING.*

**Matronship of Boys' School.**

To those people who are really fond of boys the post of matron in a boys' school should be most interesting and congenial. If such a post embraces the charge of a sanatorium, it is usually filled by a trained nurse. Sometimes, of course, the post is merely that of a housekeeper and needlewoman. Such posts are usually advertised.—Isobel.

### *WAR MATTERS.*

**Address Required.**

The address of the Uniform Department of the British Red Cross Society is Dorland House, Regent Street, S.W. 1.—Constance.

### *MISCELLANEOUS.*

**X-Ray Bags.**

William Tylar, of 20 Redcar Road, N.S., Blackpool, supply the yellow and black light-tight *x*-ray bags for carrying plates before and after exposure. They are made up in packets of twelve yellow and twelve black bags, and the prices are: for $\frac{1}{4}$-plate size, 1s. 3d.; $\frac{5}{4}$, 1s. 6d; $\frac{1}{2}$, 3s.; $\frac{1}{1}$, 2s. 9d.; $\frac{1}{8}^{0}$, 4s. 6d.; $\frac{1}{1}\frac{2}{0}$, 7s. 6d.; and $\frac{1}{1}\frac{5}{2}$, 12s.—X-Ray.

**Metol Poisoning.**

Many radiographers' assistants who are constantly developing plates suffer acute irritation of the skin of the hands as a result of continual immersion in a developing solution containing metol which, in addition to other chemical salts, acts as an irritant poison to which some skins are especially susceptible. An excellent preparation is now on the market prepared by Peat Products (Sphagnol), Ltd., 18 and 19 Queenhithe, London, in the form of medical soap and ointment, which is especially useful for treating cases of this character. It can be obtained through most photographic chemists.—Assistant.

**Rubber Sheeting.**

We are of opinion that the mistake you make in the purchase of rubber goods such as sheeting and water-beds is that you economise in price at the expense of quality. All rubber goods are exceedingly expensive now, but the best class, while costing 10 to 20 per cent. more, will last more than double the time. A really good water-bed, with the use it gets in hospital, should, with proper care, last at least five years. The cheap quality will break down in a year, or at the most two years. Our experience of Warne's rubber goods leads us to recommend them. Messrs. Albert Browne, of Leicester, deal largely in them.—K. T. E.

**X-Ray Paper and its Value.**

Although prints from *x*-ray plates can never be so useful for diagnostic purposes as the plate itself, it is nevertheless very important that prints be taken from these plates which can be used as a record for transmission from place to place. This is especially important in military work, and many of the hospitals abroad as well as hospitals in this country make it a practice to transmit with the case papers the print of such radiograms as have been taken. The choice, therefore, of a suitable paper is of considerable importance, and preference will necessarily be given to that type which will reproduce the most perfect detail. Our experience of the special *x*-ray papers prepared by Illingworth and Co., Ltd., is especially pleasing. Their contrast bromides, when used with suitable plates, give brilliant results and are exceptionally easy to manipulate. One of the greatest difficulties of printing successfully from *x*-ray plates is due to the fact that they are very strong in contrasts. It is impossible without special paper to reproduce some plates, for the exposure necessary to reproduce detail in one part may be sufficient to blot out detail in another. In these cases Illingworth Gas-light Paper is exceedingly useful, for after giving an initial exposure it may be shaded by passing a piece of opaque card between the plate and the source of light from the dense to the thin part of the plate. This process permits of the perfect reproduction of the whole plate. The address of Messrs. T. Illingworth and Co., Ltd., is Park Royal, Willesden Junction, London, N.W. 10.

